# Editorial: Malnutrition: A Cause or a Consequence of Poverty?

**DOI:** 10.3389/fpubh.2021.796435

**Published:** 2022-01-04

**Authors:** Zheng Feei Ma, Chee Woon Wang, Yeong Yeh Lee

**Affiliations:** ^1^Department of Health and Environmental Sciences, Xi'an Jiaotong-Liverpool University, Suzhou, China; ^2^Department of Biochemistry, Faculty of Medicine, MAHSA University, Jenjarom, Malaysia; ^3^School of Medical Sciences, Universiti Sains Malaysia, Kota Bharu, Malaysia

**Keywords:** malnutrition, public health, global health, poverty, inequality

Malnutrition is one of the universal public health issues affecting populations worldwide. In addition, it is an obstacle to the eradication of the poverty. Therefore, it is estimated that with the elimination of malnutrition, about 32% of the worldwide disease burden could be removed (1). The 2030 Agenda for Sustainable Development and its 17 Sustainable Development Goals (SDGs) has provided a framework to promote sustainable development (2). One of the SDGs, SDG 2, is to “end hunger, achieve food security and improved nutrition, and promote sustainable agriculture” (2). Malnutrition can be categorized into undernutrition (e.g., wasting, stunting, and underweight), overnutrition (e.g., obesity), and micronutrient-related malnutrition (3). Globally, it is estimated that 149 million children are stunted and 50 million are wasted (4). Undernutrition has been reported to be accounted for 45% of all child deaths either as a direct or underlying cause (5). Also, an estimated of 40 million children under 5 years are considered overweight and 678 million adults are categorized to be obese (4). The first 1,000 days of life has been targeted as the window period to address malnutrition because malnutrition manifestation and symptoms usually begin to appear during this period (6). Therefore, the collection of the published papers in this Research Topic aimed to focus on nutrition education, social determinants of health, factors, and consequences of malnutrition, and suggestions to prevent malnutrition in populations (Alves et al.; Jansen et al.; le Roux et al.; Siddiqui et al.).

Malnutrition and poverty have become two sides of a same coin that are ravaging the developing countries, especially in the African continent (7). Although poverty has reported to be the main cause of malnutrition, malnutrition at an early age can deepen the influence of poverty and entrap these malnourished individuals in the “cycle of poverty” with potentially serious and long lasting health consequences (8). Studies have reported that both environmental and dietary factors are one of the causes of the malnutrition, especially in children. Diet and diseases are considered the primary determinants, with access to health care facilities, healthy food and physical environment, nutrition knowledge, feeding practices, education level, household income, and household food security influenced by the socio-demographics (9).

However, it is very challenging to determine if malnutrition can be considered a cause or consequence of poverty (Siddiqui et al.). Individuals living in poverty have limited access to necessities such as clean water, hygiene, and healthy food products. The consequences of poverty on individuals include food insecurity, poor health, and nutritional status (Siddiqui et al.). On the other hand, when nutritional status of individuals is improved, good health can be achieved. Consequently, this can contribute to increased work productivity levels of individuals, which can potentially lead to increased income of individuals (Siddiqui et al.). Poverty is associated with hidden hunger and nutrient deficiencies, which exacerbate the severity of malnutrition in individuals. Therefore, both malnutrition and poverty seem to reinforce each other as a vicious cycle (Siddiqui et al.).

The COVID-19 pandemic has exacerbated the severity of malnutrition issue, especially for the individuals susceptible to poverty (10). Some of the impacts of the COVID-19 pandemic on the food systems include the restrictions on movement of food products, food price volatility, and disruptions on food supply chain. In addition, due to the COVID-19 pandemic, the reduction in household income has affected the ability of the household to purchase food, which further compounded the food security issue among individuals living in poverty. The prevalence of wasting has been predicted to increase by 14% due to the COVID-19 pandemic (11). There have been increasingly studies that reported the importance of adequate nutrition in resilience to infection and as a mediator of its effects (12, 13). Sufficient dietary intake of macronutrients and micronutrients are essential for maintaining proper immune functioning (14). Undernutrition, especially in children can potentially lead to immune dysfunction and increased susceptibility to infectious diseases. Overnutrition can develop into overweight and obesity, which increases the risk of developing its comorbidities, such as cardiovascular disease (CVD) and diabetes. These comorbidities have been reported to be known risk factors for COVID-19 because they can increase the risk of SARS-CoV-2 infection and developing severe COVID-19 complications (15–17).

## Call to Action

Several solutions have been proposed to address the burden of malnutrition and poverty. These include: healthy food and physical environment, access to basic services (e.g., wash, sanitation, hygiene, and health care), diverse and nutritious food products, and exclusive breastfeeding for the first 6 months of life. In addition, all nutrition experts and policy makers must work together to strengthen the government agencies that focuses on nutrition policy and address urgent nutrition problems across the life cycle including infants, children, elderly, pregnant, and lactating women (18). Data on nutrition status of vulnerable populations should be routinely collected (18). Health economists should perform cost analyses on nutrition care, which translate nutrition policies into health care practice by the government (18). Synergistic collaboration between stakeholders in nutrition sectors should be encouraged to meet local and national nutrition goals set by the government (18). Nutrition education programmes should be developed and promoted to the general population to increase their awareness of healthier dietary choices (18).

Therefore, combating malnutrition, coupled with the COVID-19 pandemic, is one of the greatest global health challenges in the twenty-first century, which has disproportionally affected vulnerable groups. In addition, there is a need to build a sustainable and resilient food system that can provide adequate food supplies for all. Given the relationship between malnutrition and poverty, improved nutrition can help to lay the foundation for a stable population with progress made in education, health, and women empowerment because poverty and social inequality are reduced.

## Author Contributions

ZFM drafted and wrote the first draft of the manuscript. All authors reviewed and revised the manuscript, agreed to be accountable for all aspects of the work, read, and approved the final manuscript.

## Conflict of Interest

The authors declare that the research was conducted in the absence of any commercial or financial relationships that could be construed as a potential conflict of interest.

## Publisher's Note

All claims expressed in this article are solely those of the authors and do not necessarily represent those of their affiliated organizations, or those of the publisher, the editors and the reviewers. Any product that may be evaluated in this article, or claim that may be made by its manufacturer, is not guaranteed or endorsed by the publisher.
